# Pairwise Accelerated Failure Time Regression Models for Infectious Disease Transmission in Close‐Contact Groups With External Sources of Infection

**DOI:** 10.1002/sim.10226

**Published:** 2024-10-03

**Authors:** Yushuf Sharker, Zaynab Diallo, Wasiur R. KhudaBukhsh, Eben Kenah

**Affiliations:** ^1^ Data Sciences Institute Takeda Pharmaceuticals USA Cambridge Massachusetts USA; ^2^ Biostatistics Division, College of Public Health The Ohio State University Columbus Ohio USA; ^3^ School of Mathematical Sciences University of Nottingham Nottingham UK

**Keywords:** accelerated failure time model, infectious disease epidemiology, secondary attack risk, survival analysis

## Abstract

Many important questions in infectious disease epidemiology involve associations between covariates (e.g., age or vaccination status) and infectiousness or susceptibility. Because disease transmission produces dependent outcomes, these questions are difficult or impossible to address using standard regression models from biostatistics. Pairwise survival analysis handles dependent outcomes by calculating likelihoods in terms of contact interval distributions in ordered pairs of individuals. The contact interval in the ordered pairij is the time from the onset of infectiousness ini to infectious contact fromi toj, where an infectious contact is sufficient to infectj if they are susceptible. Here, we introduce a pairwise accelerated failure time regression model for infectious disease transmission that allows the rate parameter of the contact interval distribution to depend on individual‐level infectiousness covariates fori, individual‐level susceptibility covariates forj, and pair‐level covariates (e.g., type of relationship). This model can simultaneously handle internal infections (caused by transmission between individuals under observation) and external infections (caused by environmental or community sources of infection). We show that this model produces consistent and asymptotically normal parameter estimates. In a simulation study, we evaluate bias and confidence interval coverage probabilities, explore the role of epidemiologic study design, and investigate the effects of model misspecification. We use this regression model to analyze household data from Los Angeles County during the 2009 influenza A (H1N1) pandemic, where we find that the ability to account for external sources of infection increases the statistical power to estimate the effect of antiviral prophylaxis.

## 1 Introduction

Many important questions in infectious disease epidemiology involve the effects of covariates (e.g., age or vaccination status) on the risk of transmission. Longitudinal studies of households and other groups of close contacts are one of the most valuable sources of information about mechanisms and risk factors for transmission [1, 2]. These studies have been used to understand many recent emerging and re‐emerging epidemics, including pertussis [3], SARS coronavirus [4], 2009 pandemic influenza A (H1N1) [5, 6, 7, 8, 9], MERS coronavirus [10], Ebola virus disease [11, 12], norovirus [13, 14], hand‐foot‐and‐mouth disease [15], cryptosporidium [16], measles [17], and COVID‐19 [18, 19]. Almost all of these studies are analyzed using logistic regression or other standard models for binary outcomes. These implicitly attribute all subsequent infections in the household to the primary case, failing to account for multiple generations of transmission within the household and for the ongoing risk of infection from sources outside the household [20].

The transmission of disease creates dependent outcomes in different individuals because the infection of one individual alters the risk of infection in other individuals. These dependencies are not accounted for in standard regression models, which assume independent or conditionally independent outcomes in different individuals [21]. Attempts to account for these dependencies using robust variance are ineffective because they do not address the bias in the underlying point estimates [20]. When exposure to infection differs systematically in susceptible individuals with different covariate values, estimates of covariate effects on susceptibility that do not account for this dependence can be biased even in randomized trials [22, 23].

In pairwise survival analysis, dependent outcomes in individuals are handled by analyzing failure times in ordered pairs of individuals [24, 25]. In the ordered pairij, the *contact interval* is the time from the onset of infectiousness ini to infectious contact fromi toj, where an infectious contact is defined to be a contact sufficient to infectj if they are susceptible. The survival function of the contact interval distribution can be used to calculate the probability of infectious contact fromi toj over any given time interval during whichi is infectious. The probability of infectious contact withj during the entire infectious period ofi is called the *secondary attack risk* (SAR). The hazard function of the contact interval distribution is an estimate of the *infectiousness profile*, which is the relative infectiousness ofi as a function of time elapsed since the onset of infectiousness. The contact interval fromi toj is right‐censored if the infectious period ofi ends prior to infectious contact withj, ifj is infected from a source other thani, or if observation of the pairij ends beforej is infected. When who‐infected‐whom is observed, standard parametric and nonparametric methods from survival analysis can be used to estimate the contact interval distribution. When who‐infected‐whom is not observed, parametric likelihoods can be integrated over all possible transmission trees [25] or nonparametric estimates from all possible transmission trees can be averaged using an expectation‐maximization algorithm [26].

For transmission from an infectious individuali to a susceptible individualj, there are three possible types of covariates: individual‐level covariates fori could affect their infectiousness, individual‐level covariates forj could affect their susceptibility, and pair‐level covariates (e.g., type of relationship) could affect the risk of transmission independently of the infectiousness ofi or the susceptibility ofj. Estimation of these effects can be used to design and evaluate public health responses to epidemics [27, 28].

To allow semiparametric estimation of covariate effects on the hazard of transmission, Kenah [29] developed a regression model in which the hazard of infectious contact fromi toj was

(1) $$h_{ij}(\tau )=e^{\beta^{⊤}X_{ij}}h_{0}(\tau )$$

whereβ is an unknown coefficient vector,$h_{0}(\tau )$ is an unspecified baseline hazard for the contact interval, and$X_{ij}$ is a vector that can include individual‐level infectiousness covariates fori, individual‐level susceptibility covariates forj, and pair‐level covariates. Although it produces consistent and asymptotically normal estimates ofβ, this model assumes that all transmissions occur between individuals under observation. This inability to account for external sources of infection is a fundamental limitation that must be addressed before pairwise survival analysis can become a practical tool for infectious disease epidemiology.

In this article, we develop a pairwise accelerated failure time (AFT) regression model that allows the rate parameter of the contact interval distribution to depend on covariates while accounting simultaneously for the risk of infection from internal and external sources. We use the theory of counting processes and a simulation study to show that parameter estimates from this model are consistent and asymptotically normal. Our simulation study also highlights the critical role of epidemiologic study design in parameter estimation and investigates the effects of model misspecification. We apply the pairwise AFT regression model to household surveillance data collected by the Los Angeles County Department of Public Health during the 2009 influenza A (H1N1) pandemic, and we find that accounting for external infection improves statistical power to estimate the effect of antiviral prophylaxis. The pairwise AFT model has the potential to become an important new statistical tool in infectious disease epidemiology, with potential applications that include the design and analysis of vaccine trials, outbreak investigations, and the analysis of contact tracing data or household studies.

### 1.1 Stochastic S(E)IR Models

The pairwise AFT regression model is based on a general stochastic model of transmission. At any time, each individuali∈{1,…,n} is in one of four states: susceptible (S), exposed (E), infectious (I), or removed (R). Personi moves from S to E at their *infection time* $t_{i}$, with$t_{i}=\infty$ ifi is never infected. After infection,i has a *latent period* of length$\varepsilon_{i}$ during which they are infected but not infectious. At time$t_{i}+\varepsilon_{i}$,i moves from E to I, beginning an *infectious period* of length$\iota_{i}$. At time$t_{i}+\varepsilon_{i}+\iota_{i}$,i moves from I to R, after which they can no longer infect others or be infected. The latent period$\varepsilon_{i}$ is a nonnegative random variable, and the infectious period$\iota_{i}$ is a strictly positive random variable. We assume that the latent and infectious periods both have finite mean and variance. The time elapsed since the onset of infectiousness ini at time$t_{i}+\varepsilon_{i}$ is called the *infectious age* ofi. An SIR model is an SEIR model with latent period$\varepsilon_{i}=0$ for alli.

After becoming infectious at time$t_{i}+\varepsilon_{i}$, personi makes infectious contact withj≠i at time$t_{ij}=t_{i}+\varepsilon_{i}+\tau_{ij}^{*}$. The *infectious contact interval* $\tau_{ij}^{*}$ is a strictly positive random variable with$\tau_{ij}^{*}=\infty$ if infectious contact never occurs. Because infectious contact can only occur whilei is infectious, we always have$\tau_{ij}^{*}\in (0,\iota_{i}]$ or$\tau_{ij}^{*}=\infty$. Because we define infectious contact to be sufficient to infect a susceptible person,$t_{j}\leq t_{ij}$ for alli andj.

An *internal infection* occurs when an individual is infected by another individual in the observed population. For each ordered pairij, let$C_{ij}=1$ if infectious contact fromi toj is possible and$C_{ij}=0$ otherwise. For example,$C_{ij}$ could be an entry in the adjacency matrix for a contact network through which infection is transmitted. We do not assume that$C_{ij}=C_{ji}$, so this network could contain directed edges. For simplicity, we have written$C_{ij}$ as a constant. However, our methods extend to time‐varying contact patterns where$C_{ij}$ is a function of time (e.g., a dynamic contact network).

**FIGURE 1:**
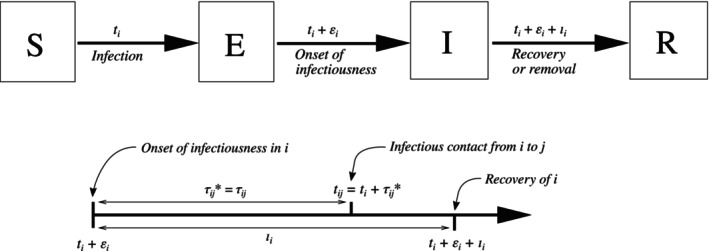
Notation for the stochastic SEIR model natural history (top) and infectious contact process (bottom) [29]. Here, we have$\tau_{ij}^{*}=\tau_{ij}$ because$\tau_{ij}\leq \iota_{i}$. Otherwise, we would have$\tau_{ij}^{*}=\infty$ and$t_{ij}=\infty$ so infectious contact fromi toj never occurs.

The infectious contact interval$\tau_{ij}^{*}$ is generated as follows: a *contact interval* $\tau_{ij}$ is drawn from a failure time distribution with hazard function$h_{ij}(\tau )$. If$\tau_{ij}\leq \iota_{i}$ and$C_{ij}=1$, then$\tau_{ij}^{*}=\tau_{ij}$. Otherwise,$\tau_{ij}^{*}=\infty$. The hazard function$h_{ij}(\tau )$ is the instantaneous infectiousness ofi at timeτ after the onset of infectiousness. The cumulative hazard function is$H_{ij}(\tau )=\int_{0}^{\tau }h_{ij}(u)\;du$, and the secondary attack risk is$1-S_{ij}(\iota_{i})$, where$S_{ij}(\tau )=\exp(-H_{ij}(\tau ))$ is the survival function. Figure 1 illustrates an example trajectory in individuali and an infectious contact fromi toj.

An *external infection* occurs when an individual is infected from a source outside the observed population, such as an environmental source or a community source (i.e., an individual who is not under observation). Let$C_{0j}$ indicate whether individualj is at risk of external infectious contact. Let the *external infectious contact time* $t_{0j}^{*}$ denote the first time that an individualj receives infectious contact from outside the observed population, with$t_{0j}^{*}=\infty$ if this never occurs. We assume that the external infectious contact time is generated as follows: an *external contact interval* $\tau_{0j}$ is drawn from a failure time distribution with hazard function$h_{0j}(t)$. If$C_{0j}=1$, then$t_{0j}^{*}=\tau_{0j}$. Otherwise,$t_{0j}^{*}=\infty$. For simplicity, we assume the external source is always infectious so$t_{0}=\varepsilon_{0}=0$ and$\iota_{0}=\infty$. This assumption could be relaxed by defining an infectiousness onset time$t_{0}+\varepsilon_{0}$ and infectious period$\iota_{0}$ for the external source.

### 1.2 Exposure and Infectious Sets

For each internal infectionj, let$v_{j}$ denote the index of his or her infector. Let$v_{j}=0$ ifj is an external infection and$v_{j}=\infty$ ifj is not infected. When$v_{j}$ is observed for all infectedj, we say that *who‐infected‐whom* is observed. Otherwise, we say that who‐infected‐whom is not observed even if$v_{j}$ is observed for a subset of infectedj. The *exposure set* of an individualj is

(2) $${𝒲}_{j}=\{i<\infty :(t_{i}+\varepsilon_{i}<t_{j}\;\text{or}\;i=0)\;\text{and}\;C_{ij}=1\}$$

which is the set of all sources of infection to whomj was exposed while susceptible. The *infectious set* of individualj is the set of all possible$v_{j}$, which we denote${𝒱}_{j}$. Given$C_{ij}$ and transfer times between the states (S, E, I, and R), we must have

(3) $${𝒱}_{j}\subseteq \{i<\infty :(t_{i}+\varepsilon_{i}<t_{j}\leq t_{i}+\varepsilon_{i}+\iota_{i}\;\text{or}\;i=0)\;\text{and}\;C_{ij}=1\}$$

If the infector ofj is known, then${𝒱}_{j}=\{v_{j}\}$. Ifj was not infected, then${𝒱}_{j}=Ø$ (the empty set).

### 1.3 Infectious Disease Data

The contact interval$\tau_{ij}$ can be observed only ifj is infected byi at time$t_{ij}=t_{i}+\varepsilon_{i}+\tau_{ij}$. This can happen only if$C_{ij}=1$ and the pairij is at risk of transmission at time$t_{ij}$. Contact intervals can be right‐censored by the end of infectiousness ini, by the infection ofj from a source other thani, or by the end of observation. Fori≠0, let${ℐ}_{i}(t)={𝕀}_{t-t_{i}-\varepsilon_{i}\in (0,\iota_{i}]}$ indicate whetheri remains infectious at timet, and let${ℐ}_{0}(t)$ indicate whether external infectious contact is possible at timet. Let${𝒮}_{j}(t)={𝕀}_{t\leq t_{j}}$ indicate whetherj remains susceptible at timet, and let$𝒪(t)={𝕀}_{t\leq T}$ indicate whether observation is ongoing at timet. Since${ℐ}_{i}(t)$,${𝒮}_{j}(t)$, and𝒪(t) are right‐continuous, the process

(4) $$Y_{ij}(t)=C_{ij}{ℐ}_{i}(t){𝒮}_{j}(t)𝒪(t)$$

is right‐continuous and equals one when there is a risk of an observed infectious contact fromi toj at timet. The assumptions made in the stochastic S(E)IR model above ensure independent right censoring and left truncation of$\tau_{ij}$ and$\tau_{0j}$.

Our epidemiologic data contain the times of allS→E (infection),E→I (infectiousness onset), andI→R (removal) transitions in the observed population between time 0 and a timeT that is a stopping time with respect to the observed data. For all ordered pairsij in whichi is infected ori=0, we observe$C_{ij}$. For each ordered pairij that was at risk of an observed transmission fromi toj, we need a starting time when this risk began, a stopping time when this risk ends, and an event indicator$\delta_{ij}$ that equals onei is a possible infector ofj (i.e.,$i\in {𝒱}_{j}$) and zero otherwise. As we explain below, the data for each pairij can contain individual‐level covariates fori, individual‐level covariates forj, and pair‐level covariates.

## 2 Methods

Although any parametric failure time distribution could be used in this model, we focus on the following three because they have simple closed‐form survival and hazard functions: the exponential distribution with rateλ, the Weibull distribution with rateλ and shapeγ, and the log‐logistic distribution with rateλ and shapeγ. The internal and external transmission models can use the same failure time distribution or different distributions. Let the parameters of the internal failure time distribution be$\left(\lambda_{\text{int}},\gamma_{\text{int}}\right)$ and the parameters of the external distribution be$\left(\lambda_{\text{ext}},\gamma_{\text{ext}}\right)$, where the shape parameters are omitted for the exponential distribution.

The internal and external transmission models generally work on different time scales (i.e., with different time origins). In a pairij withi≠0, the time origin is the onset of infectiousness ini, which can differ from pair to pair. A pair0j is at risk of transmission whenj is susceptible and external infectious contact is possible. Typically, a common time origin will be specified for all external pairs in a single population under observation.

### 2.1 Internal and External Rate Parameters

Wheni≠0, the rate parameter of the contact interval distribution in the pairij is

(5) $$\lambda_{ij}=e^{\beta_{\text{int}}^{⊤}X_{ij}}\lambda_{0}$$

where$\beta_{\text{int}}$ is an unknown coefficient vector,$\lambda_{0}$ is a baseline rate parameter, and$X_{ij}$ is a covariate vector that can include infectiousness covariates fori, susceptibility covariates forj, and pair‐level covariates. Each component of$\beta_{\text{int}}$ is the log rate ratio for a one‐unit increase in the corresponding covariate while holding all others constant. Because$\lambda_{0}>0$, it will be estimated using an intercept$\ln\lambda_{0}$. This model is equivalent to an AFT model where$\exp(-\beta_{\text{int}}^{T}X_{ij})$ is the acceleration factor [30]. We prefer to define the model in terms of rate ratios because the rate ratio is a more common measure of association in epidemiology [31, 32].

The rate parameter for the external contact interval for individualj is

(6) $$\lambda_{0j}=e^{\beta_{\text{ext}}^{⊤}X_{0j}}\mu_{0}$$

where$\beta_{\text{ext}}$ is an unknown coefficient vector, and$\mu_{0}$ is the baseline external rate parameter, and$X_{0j}$ is a covariate vector that can include susceptibility covariates forj and environmental or community covariates. Like$\beta_{\text{int}}$, the components of$\beta_{\text{ext}}$ are log rate ratios and estimation of$\mu_{0}$ will be done using an intercept$\ln\mu_{0}$.

There may be overlap between the internal coefficient vector$\beta_{\text{int}}$ and the external coefficient vector$\beta_{\text{ext}}$. For example, vaccination status could affect the rate parameters for both models. To handle this, we parameterize the combined model as

(7) $$\lambda_{ij}=e^{\beta^{⊤}X_{ij}}\lambda_{0}^{1-{𝕀}_{i=0}}\mu_{0}^{{𝕀}_{i=0}}$$

where the coefficient vectorβ includes coefficients unique to the internal model, coefficients unique to the external model, and shared coefficients. The components of$X_{ij}$ used only in the internal model are set to zero wheni=0, and the components of$X_{ij}$ used only in the external model are set to zero wheni≠0. The distinction between internal and external rows in the data set is maintained using an *external pair indicator* $\zeta ={𝕀}_{i=0}$. If a covariate in$X_{ij}$ is shared by the internal and external transmission models, it can be allowed to have different coefficients in the two models by including an interaction term withζ. We call these *external interaction terms*. The parameter vector$X_{ij}$ can include time‐varying covariates, which are handled in the same way as in standard survival analysis [30, 33].

### 2.2 Maximum Likelihood Estimation

The likelihood and its score process can be derived in a manner similar to that of Kenah [25]. Letθ be a coefficient vector containing the log rate ratiosβ, the log baseline rate parameters$\ln\lambda_{0}$ and$\ln\mu_{0}$, and the log shape parameters$\ln\gamma_{\text{int}}$ and$\ln\gamma_{\text{ext}}$ as needed. Let$h_{ij}(t,\theta )$ and$S_{ij}(t,\theta )$ be the hazard and survival functions for the contact interval distribution with rate$\lambda_{ij}$ from Equation (7). The parametric family may be different fori≠0 andi=0, which is implemented using the external row indicatorζ. Let$\theta_{0}$ denote the true value ofθ.

#### 2.2.1 Who‐Infected‐Whom Observed

Let${𝒩}_{ij}(t)={𝕀}_{t\geq t_{ij}}$ count the first infectious contact fromi toj. Assumej is susceptible at timet=0, so${𝒩}_{ij}(0)=0$. Then${ℳ}_{ij}(t,\theta_{0})$ is a mean‐zero martingale, where

(8) $${ℳ}_{ij}(t,\theta )={𝒩}_{ij}(t)-\int_{0}^{t}h_{ij}(u-t_{i}-\varepsilon_{i},\theta )C_{ij}{ℐ}_{i}(u)\;du$$

and we let$t_{0}=\varepsilon_{0}=0$. We observe infectious contacts fromi toj only whilej is still susceptible and the pairij is under observation, which gives us the observed counting process

(9) $$N_{ij}(t)=\int_{0}^{t}Y_{ij}(u)\;d{𝒩}_{ij}(u)$$

Similarly, let

(10) $$M_{ij}(t,\theta )=\int_{0}^{t}Y_{ij}(u)\;d{ℳ}_{ij}(u,\theta )$$

Then$M_{ij}(t,\theta_{0})$ is a mean‐zero martingale because it is the integral of a predictable process with respect to the mean‐zero martingale${ℳ}_{ij}(u,\theta_{0})$.

When we observe infectious contacts fromi toj between time 0 and timeT, we get the log likelihood

(11) $$\begin{array}{ll} \ell_{ij}^{*}(\theta ) & =\int_{0}^{T}\ln h_{ij}(u-t_{i}-\varepsilon_{i},\theta )\;dN_{ij}(u)\\ & \;-\int_{0}^{T}h_{ij}(u-t_{i}-\varepsilon_{i},\theta )Y_{ij}(u)\;du \end{array}$$

This is a standard log likelihood for right‐censored and left‐truncated data: the first term is a log hazard ifi infectsj and zero otherwise, and the second term is the negative cumulative hazard of infectious contact. The score process is

(12) $$U_{ij}^{*}(t,\theta )=\int_{0}^{t}\left(\frac{\partial }{\partial \theta }\ln h_{ij}(u-t_{i}-\varepsilon_{i},\theta )\right)\;dM_{ij}(u,\theta )$$

which is a mean‐zero martingale when$\theta =\theta_{0}$.

Now fixj. If we observe all pairsij from time 0 until timeT, the log likelihood is

(13) $$\ell_{\cdot j}^{*}(\theta )=\underset{i:i\neq j}{\sum }\ell_{ij}^{*}(\theta )$$

with score process

(14) $$U_{\cdot j}^{*}(t,\theta )=\underset{i:i\neq j}{\sum }U_{ij}^{*}(t,\theta )$$

which is a mean‐zero martingale because it is a sum of mean‐zero martingales.

When we observe who‐infected‐whom, the log likelihood is$\ell^{*}(\theta )=\sum_{j=1}^{n}\ell_{\cdot j}^{*}(\theta )$ and its score process is$U^{*}(t,\theta )=\sum_{j=1}^{n}U_{\cdot j}^{*}(t,\theta )$. Because it is a sum of mean‐zero martingales,$U^{*}(t,\theta_{0})$ is also a mean‐zero martingale. Differentiating$\ell^{*}(\theta )$ twice, evaluating at$\theta_{0}$, and taking expectations yields

(15) $$𝔼[-\frac{\partial^{2}}{\partial \theta^{2}}\;\ell^{*}(\theta )\;{|}_{\theta =\theta_{0}}]=𝔼[\langle U^{*}(\theta_{0})\rangle (T)]$$

where$\left\langle U^{*}(\theta_{0})\rangle (t\right)$ is the predictable variation process of$U^{*}(t,\theta_{0})$. Under our regularity conditions, this implies that the variance of the maximum likelihood estimator$\hat{\theta }$ can be estimated consistently using the observed information.

#### 2.2.2 Who‐Infected‐Whom Not Observed

When who‐infected‐whom is not observed, we cannot see each$N_{ij}(t)$. Instead, we see$N_{\cdot j}(t)=\sum_{i\neq j}N_{ij}(t)$. The total hazard of infectious contact withj at timet is

(16) $$h_{\cdot j}(t,\theta )=\underset{i:i\neq j}{\sum }h_{ij}(t-t_{i}-\varepsilon_{i},\theta )C_{ij}{ℐ}_{i}(t)$$

so the process

(17) $$M_{\cdot j}(t,\theta )=N_{\cdot j}(t)-\int_{0}^{t}h_{\cdot j}(u,\theta ){𝒮}_{j}(u)𝒪(t)\;du=\underset{i\neq j}{\sum }M_{ij}(t,\theta )$$

is a mean‐zero martingale when$\theta =\theta_{0}$. Whenj is observed from time 0 to timeT, the log likelihood is

(18) $$\ell_{\cdot j}(\theta )=\int_{0}^{T}\ln h_{\cdot j}(u,\theta )\;dN_{\cdot j}(u)-\int_{0}^{T}h_{\cdot j}(u,\theta )S_{j}(u)\;du$$

and its score process is

(19) $$U_{\cdot j}(t,\theta )=\int_{0}^{t}\left(\frac{\partial }{\partial \theta }\ln h_{\cdot j}(u,\theta )\right)\;dM_{\cdot j}(u,\theta )$$

which is a mean‐zero martingale when$\theta =\theta_{0}$.

The complete‐data log likelihood when we do not observe who‐infected‐whom is$\ell (\theta )=\sum_{j=1}^{n}\ell_{\cdot j}(\theta )$ with score process$U(t,\theta )=\sum_{j=1}^{n}U_{\cdot j}(t,\theta )$. Because it is a sum of mean‐zero martingales,$U(t,\theta_{0})$ is also a mean‐zero martingale. Differentiatingℓ(θ) twice, evaluating at$\theta_{0}$, and taking expectations yields

(20) $$𝔼[-\frac{\partial^{2}}{\partial \theta^{2}}\;\ell (\theta )\;{|}_{\theta =\theta_{0}}]=𝔼[\langle U(\theta_{0})\rangle (T)]$$

where$\left\langle U(\theta_{0})\rangle (t\right)$ is the predictable variation process of$U(t,\theta_{0})$. Under our regularity conditions, this implies that the variance of$\hat{\theta }$ can be estimated consistently using the observed information.

#### 2.2.3 Pairwise Asymptotics, Optimization, and Inference

The arguments above establish the consistency and asymptotic normality of the maximum likelihood estimator$\hat{\theta }$ as the number of observed infectionsm→∞ as long as the rate of increase in the number of susceptibles at risk of infection is at least as fast as the rate of increase in the number of pairs at risk of transmission [25, 29].

Maximization of the log likelihood can be done using a range of numerical methods. The TranStat package inR allows the use of any of the optimization methods available in the optim function, including Nelder‐Mead [34], BFGS (Broyden, Fletcher, Goldfarb, and Shanno) [35], and simulated annealing [36]. In analyses of simulated data, we have seen situations where one method was unstable while another method easily found a maximum of the log likelihood. These have been rare, and the choice of optimization method does not consistently affect the practical performance of the model.

Similarly, the log likelihood can be used to conduct Wald, score or likelihood ratio hypothesis tests and to calculate the corresponding confidence intervals. The TranStat package currently includes *p*‐values and confidence intervals based on Wald and likelihood ratio tests. Likelihood ratio confidence intervals are more difficult to calculate, but (as expected [37]) they perform slightly better in terms of coverage probability and width than Wald confidence intervals.

## 3 Simulations

The proposed pairwise AFT regression model was tested through2000 network‐based simulations for each of two different baseline internal contact interval distributions: exponential (λ=ln(−ln0.8)) and log‐logistic(γ=2,λ=0.5). The infectious period was fixed to one time unit, so the household secondary attack risk was 0.2 in a pair with both covariates equal to zero. In all simulations, the external contact interval distribution was exponential with rate$\lambda_{\text{ext}}=0.5\ln(-\ln0.8)$.

In each simulation, we generated an undirected network representing 300 households of size five. Each household was a complete graph of size five, and the households were not connected to each other. Once a household member was infected, other members of the household could be infected by transmission within the household or by an external source. Each epidemic was followed until 500 infections occurred, which guaranteed at least 200 infections in individuals who were not primary cases (i.e., the first case in a household).

Each individuali was assigned an independent Bernoulli(0.5) covariate$X_{i}$. The rate parameter for the contact interval distribution in the pairij was

(21) $$\lambda_{ij}=\exp(\beta_{\inf}X_{i}+\beta_{\text{sus}}X_{j}+{𝕀}_{i\neq 0}\ln\lambda_{0}+{𝕀}_{i=0}\ln\mu_{0})$$

where we set$X_{0}=0$. For each simulation, the true values of$\beta_{\inf}$ and$\beta_{\text{sus}}$ were independent samples from a uniform(−1,1) distribution.

In each simulation, we analyzed data sets under four different epidemiologic study designs. Analysis of within‐household transmission is the same for all study designs, but they differ in their inclusion of person‐time from individuals at risk of external infectious contact (i.e., pairs0j). The first two study designs are “valid” in the sense that their inclusion of pair‐time at risk of transmission includes external sources and is predictable with respect to the observed data, so they do not generate immortal time bias [38]. The valid study designs are:

**Complete cohort:** Follow‐up for all2500 individuals starts at time zero, which is the time origin for external infectious contact intervals.

**Contact tracing (CT) with delayed entry:** Follow‐up of each individual begins at the infection time of the index case in his or her household. Time at risk of external infectious contact prior to the start of follow‐up is left‐truncated, and individuals in households with no infections are excluded from the study.

The second two study designs are “flawed” in the sense that their inclusion of pair‐time at risk of transmission either generates immortal time bias or fails to include external sources of infection. The flawed study designs are:

**CT without delayed entry:** Follow‐up of all members of households where at least one infection occurs starts retroactively at time zero. Individuals in households with no infections are excluded from the study.

**Ignoring external infection:** All pairs0j are excluded from the study. This is equivalent to assuming that, in each household, all infections after the primary case are caused by within‐household transmission (but not necessarily by the primary case).

Under each of the four study designs, data were analyzed both with and without observation of who‐infected‐whom. In all eight analyses of each simulation, we obtained maximum likelihood point estimates of$\beta_{\inf}$,$\beta_{\text{sus}}$,$\ln\lambda_{0}$,$\ln\gamma_{\text{int}}$, and$\ln\mu_{0}$. For all parameters, we calculated 95% Wald confidence intervals. All regression models used an exponential distribution for external rows and a specified parametric family (exponential, Weibull, or log‐logistic) for internal rows.

The epidemic simulations were conducted at the Ohio Supercomputer Center (https://www.osc.edu) using Python version 3.9.12 with SciPy version 1.7.3 (https://www.scipy.org) [39], NetworkX version 2.7.1 (https://www.networkx.org) [40], and pandas version 1.4.2 (https://www.pandas.pydata.org) [41, 42]. The Python simulations use a network‐based epidemic simulation script called transtat_models version 0.2.0 (https://www.github.com/ekenah/transtat_models). The analysis of data from each simulated epidemic was done usingR version 4.3.0 (https://www.cran.r‐project.org) [43] with the packages survival version 3.5‐5 (https://www.github.com/therneau/survival) [44] and reticulate version 1.31 (https://www.rstudio.github.io/reticulate/) [45]. The pairwise AFT models inR are implemented in the package TranStat version 0.3.7 (https://www.github.com/ekenah/TranStat), which allows pairwise AFT models to be specified using standardR model syntax. The simulation data was analyzed on a laptop usingR version 4.3.2 [43] with the packages dplyr version 1.1.2 (https://www.dplyr.tidyverse.org) [46], stringr version 1.5.0 (https://www.stringr.tidyverse.org) [47], and xtable version 1.8‐4 (https://www.xtable.r‐forge.r‐project.org) [48]. All of these software packages are free and open‐source thanks to the work of many individuals. The Python andR code for the simulations, the simulation data, and theR code for the simulation data analysis (with instructions for use) are available in the Supporting Information.

### 3.1 Simulation Results

**FIGURE 2:**
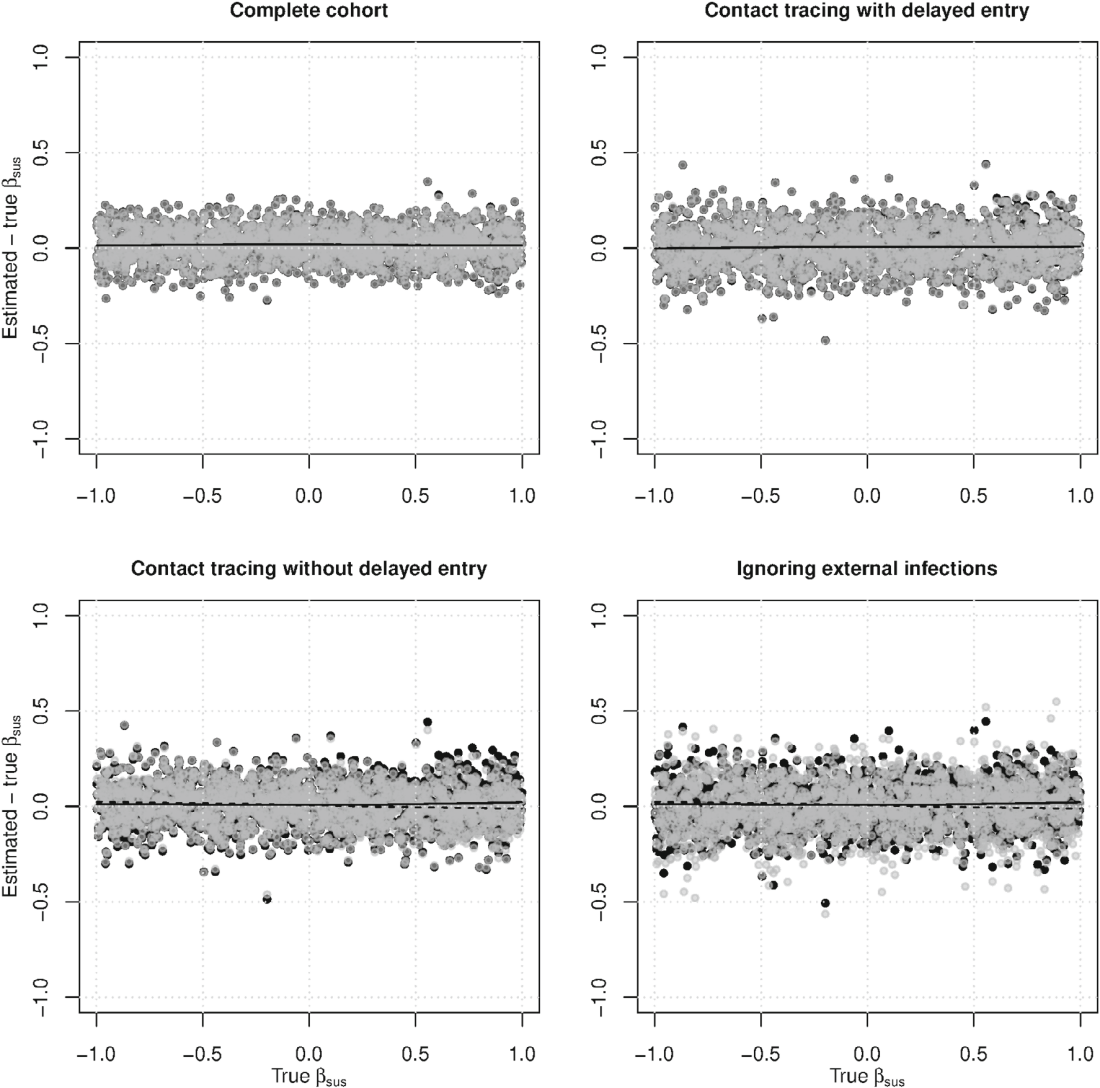
The bias${\hat{\beta }}_{\text{sus}}-\beta_{\text{sus}}$ versus the true$\beta_{\text{sus}}$ for correctly‐specified exponential pairwise AFT models fit to simulated data under all four study designs. Gray dots represent analyses where who‐infected whom was observed, and black dots represent analyses where who‐infected‐whom was not observed. In each plot, the dashed locally‐weighted polynomial regression (LOWESS) line represents the smoothed mean of the gray dots, and the solid LOWESS line represents the smoothed mean of the black dots [56]. The dashed lines are sometimes obscured by the solid lines.

Figure 2 shows scatterplots of the bias${\hat{\beta }}_{\text{sus}}-\beta_{\text{sus}}$ versus$\beta_{\text{sus}}$ for correctly‐specified pairwise AFT models fit to data generated with exponential internal contact interval distributions. In the valid study designs (top two panels), estimates when who‐infected‐whom was observed (gray dots) and estimates when who‐infected‐whom was not observed (black dots) are nearly identical, indicating that observing who‐infected‐whom makes little difference to estimation of$\beta_{\text{sus}}$. Intuitively, estimation of$\beta_{\text{sus}}$ depends mostly on who was infected, not on who infected them. In all four study designs, the smoothed means (dashed for who‐infected‐whom observed and solid for who‐infected‐whom not observed) show almost no bias across the range of$\beta_{\text{sus}}$, indicating that estimation of$\beta_{\text{sus}}$ is quite robust. A similar pattern was seen in estimates of$\beta_{\text{sus}}$ from correctly‐specified pairwise AFT models fit to data generated with log‐logistic internal contact interval distributions (see Figure S1).

**FIGURE 3:**
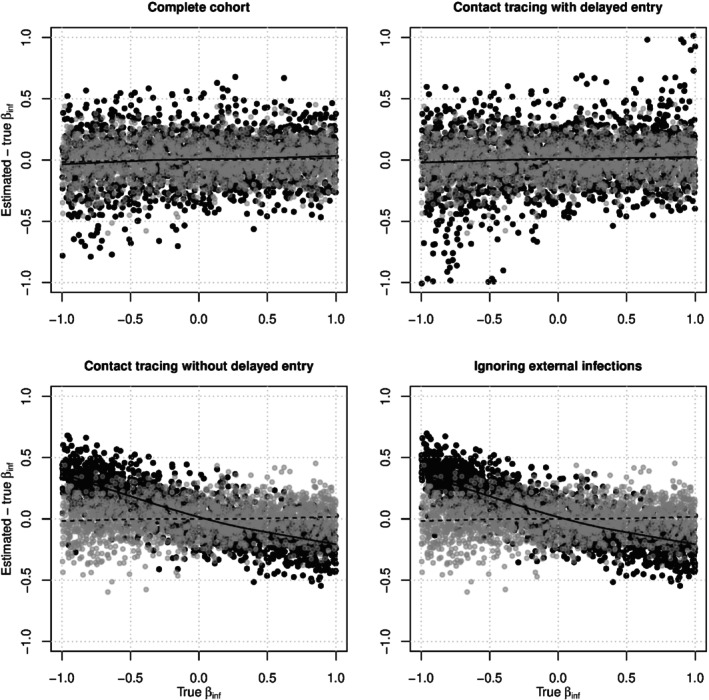
The bias${\hat{\beta }}_{\inf}-\beta_{\inf}$ versus the true$\beta_{\inf}$ for correctly‐specified exponential pairwise AFT models fit to simulated data under all four study designs. Gray dots represent analyses where who‐infected whom was observed, and black dots represent analyses where who‐infected‐whom was not observed. In each plot, the dashed LOWESS line represents the smoothed mean of the gray dots and the solid LOWESS line represents the smoothed mean of the black dots [56]. The dashed lines are sometimes obscured by the solid lines.

Figure 3 shows scatterplots of the bias${\hat{\beta }}_{\inf}-\beta_{\inf}$ versus$\beta_{\inf}$ for the same simulations. In the valid study designs (top two panels), the gray dots (who‐infected‐whom observed) have visibly smaller variance than the black dots (who‐infected‐whom not observed), indicating that observing who‐infected‐whom improves the precision of$\beta_{\inf}$ estimates. Intuitively, estimation of$\beta_{\inf}$ depends on who caused the observed infections, not just who was infected. The smoothed means from the valid study designs show almost no bias across the range of$\beta_{\inf}$ whether or not who‐infected‐whom was observed. In the flawed study designs (bottom two panels), the smoothed means show little bias when who‐infected‐whom is observed but severe bias toward the null when who‐infected‐whom is not observed. Estimating$\beta_{\inf}$ is more sensitive to epidemiologic study design than estimating$\beta_{\text{sus}}$, but information about who‐infected‐whom can make estimation of$\beta_{\inf}$ more robust. A similar pattern was seen in estimates of$\beta_{\inf}$ from correctly‐specified pairwise AFT models fit to data generated with log‐logistic internal contact interval distributions (see Figure S2).

For all parameters, Table 1 shows the coverage probabilities for Wald 95% confidence intervals for correctly‐specified exponential and log‐logistic pairwise AFT models. The valid study designs produce nominal coverage probabilities for all parameters whether or not who‐infected‐whom is observed. When who‐infected‐whom is observed, the flawed study designs produce near‐nominal coverage probabilities for$\beta_{\inf}$,$\beta_{\text{sus}}$, and the log‐logistic$\ln\gamma_{0}$ but very low coverage probabilities (or no estimates at all) for$\ln\mu_{0}$. When who‐infected‐whom is not observed, the flawed study designs produce low coverage probabilities for all parameters except$\beta_{\text{sus}}$, but the correctly‐specified log‐logistic model performed substantially better than the correctly‐specified exponential model.

**TABLE 1:** 95% confidence interval coverage probabilities for correctly‐specified pairwise AFT models with exponential and log‐logistic contact intervals.

Contact intervals	Study design	$\beta_{\text{sus}}$	$\beta_{\inf}$	$\ln\lambda_{0}$	$\ln\gamma_{\text{int}}$	$\ln\mu_{0}$
**Who‐infected‐whom observed**						
Exponential	Complete cohort	0.936	0.951	0.947	—	0.953
	CT with delayed entry	0.952	0.951	0.946	—	0.958
	*CT without delayed entry*	0.959	0.951	0.947	—	0.000
	*Ignoring external infection*	0.954	0.950	0.943	—	—
Log‐logistic	Complete cohort	0.945	0.947	0.947	0.938	0.915
	CT with delayed entry	0.949	0.948	0.944	0.936	0.960
	*CT without delayed entry*	0.950	0.949	0.943	0.936	0.000
	*Ignoring external infection*	0.948	0.947	0.941	0.939	—
**Who‐infected‐whom not observed**						
Exponential	Complete cohort	0.937	0.955	0.952	—	0.945
	CT with delayed entry	0.952	0.963	0.959	—	0.938
	*CT without delayed entry*	0.952	0.785	0.341	—	0.196
	*Ignoring external infection*	0.951	0.740	0.224	—	—
Log‐logistic	Complete cohort	0.948	0.950	0.943	0.941	0.919
	CT with delayed entry	0.954	0.948	0.936	0.934	0.915
	*CT without delayed entry*	0.949	0.929	0.911	0.716	0.894
	*Ignoring external infection*	0.950	0.928	0.911	0.711	—

*Note*: The flawed study designs are in italics.

**FIGURE 4:**
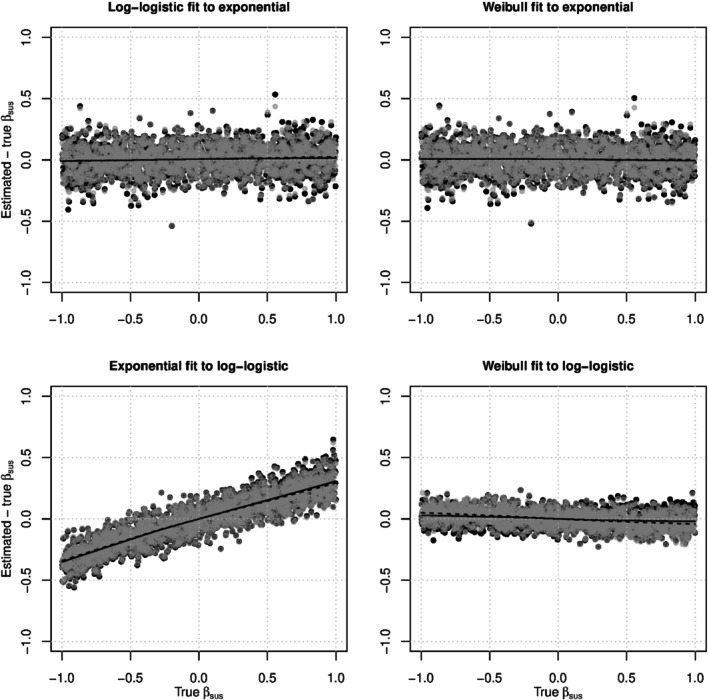
The bias${\hat{\beta }}_{\text{sus}}-\beta_{\text{sus}}$ versus the true$\beta_{\text{sus}}$ for pairwise AFT models under the contact tracing with delayed entry study design. In the top two panels, the simulated data was generated using exponential internal contact intervals, so the log‐logistic model is misspecified but the Weibull model is correctly specified. In the bottom two panels, the simulated data was generated using log‐logistic internal contact intervals, so the exponential and Weibull models are both misspecified. Gray dots represent analyses where who‐infected whom was observed, and black dots represent analyses where who‐infected‐whom was not observed. In each plot, the dashed LOWESS line represents the smoothed mean of the gray dots and the solid LOWESS line represents the smoothed mean of the black dots [56]. The dashed lines are sometimes obscured by the solid lines.

We also analyzed each set of simulation results using pairwise AFT models with (possibly) misspecified internal contact interval distributions. The top two panels of Figure 4 shows the bias${\hat{\beta }}_{\text{sus}}-\beta_{\text{sus}}$ from log‐logistic and Weibull pairwise AFT models fit to data generated with exponential internal contact intervals and the contact tracing with delayed entry design. The log‐logistic model is misspecified, but the Weibull model is correctly specified because the exponential distribution is a special case of the Weibull with shapeγ=1. The two models produced remarkably similar estimates, and the smoothed means show that both models estimated$\beta_{\text{sus}}$ with almost no bias whether (gray dots) or not (black dots) who‐infected‐whom is observed. The bottom of Figure 4 shows the bias${\hat{\beta }}_{\text{sus}}-\beta_{\text{sus}}$ from exponential and Weibull pairwise AFT models fit to data generated with log‐logistic internal contact intervals and the contact tracing with delayed entry design. The exponential model, which has only rate parameters, produced estimates that are severely biased away from the null$\beta_{\text{sus}}=0$. The Weibull model, which has rate and shape parameters, produced estimates that with only slight bias toward the null. This suggests that estimation of$\beta_{\text{sus}}$ can be robust to model misspecification when the fitted model is sufficiently flexible. In all four panels, observation of who‐infected‐whom made little difference to estimation of$\beta_{\text{sus}}$. A similar pattern was seen with a complete cohort study design (see Figure S3).

**FIGURE 5:**
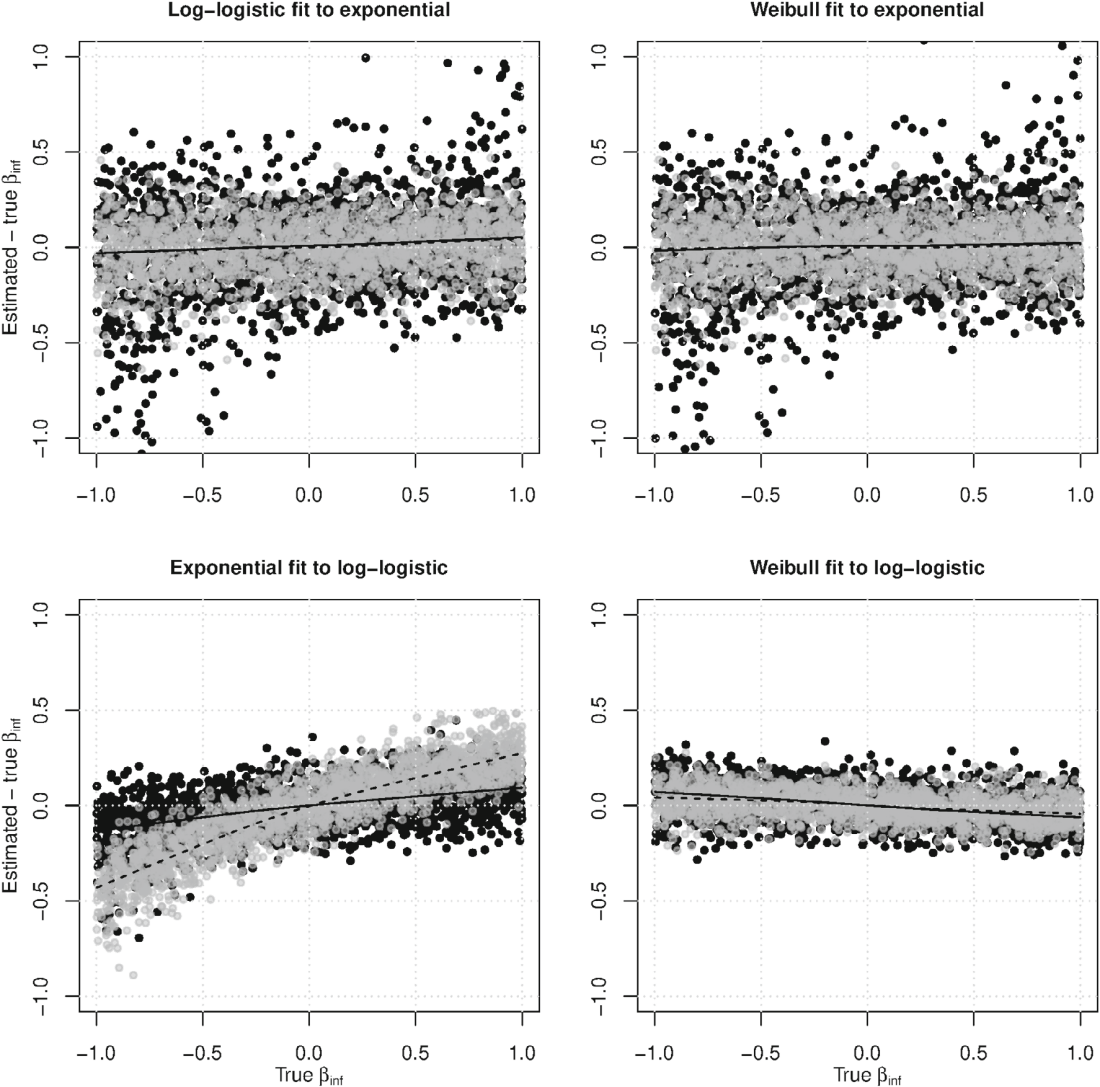
The bias${\hat{\beta }}_{\inf}-\beta_{\inf}$ versus the true$\beta_{\inf}$ for pairwise AFT models under the contact tracing with delayed entry study design. In the top two panels, the simulated data was generated using exponential internal contact intervals, so the log‐logistic model is misspecified but the Weibull model is correctly specified. In the bottom two panels, the simulated data was generated using log‐logistic internal contact intervals, so the exponential and Weibull models are both misspecified. Gray dots represent analyses where who‐infected whom was observed, and black dots represent analyses where who‐infected‐whom was not observed. In each plot, the dashed LOWESS line represents the smoothed mean of the gray dots and the solid LOWESS line represents the smoothed mean of the black dots [56]. The dashed lines are sometimes obscured by the solid lines.

The top of Figure 5 shows the bias${\hat{\beta }}_{\inf}-\beta_{\inf}$ from log‐logistic and Weibull pairwise AFT models fit to data generated with exponential internal contact intervals under the contact tracing with delayed entry design. The gray dots (who‐infected‐whom observed) have visibly smaller variance than the black dots (who‐infected‐whom not observed), indicating that observing who‐infected‐whom improves the precision of$\beta_{\inf}$ estimates. Both models estimated$\beta_{\inf}$ with almost no bias whether or not who‐infected‐whom was observed, and their estimates are remarkably similar in both cases. The bottom of Figure 5 shows estimates of$\beta_{\inf}$ from exponential and Weibull pairwise AFT models fit to data generated with log‐logistic contact intervals. Both models are misspecified, and the exponential model has one fewer parameter than a pairwise log‐logistic AFT model. The exponential model, with only rate parameters, produced estimates that are clearly biased away from the null, and the bias is clearly worse when who‐infected‐whom is observed. The Weibull model, with rate and shape parameters, produced estimates that are only slightly biased toward the null, with slightly less bias when who‐infected‐whom is observed. For estimation of log rate ratios for infectiousness, it appears to be more important that a pairwise AFT model have sufficient flexibility than it is to choose exactly the right parametric family. A similar pattern was seen with a complete cohort study design (see Figure S4).

Table 2 shows coverage probabilities from pairwise AFT models with Weibull and log‐logistic internal contact intervals analyzing simulated data generated with exponential internal contact intervals. Because the exponential distribution is a special case of the Weibull distribution, the Weibull model produces results similar to the correctly‐specified pairwise AFT models in Table 1 (except for$\ln\mu_{0}$ when who‐infected‐whom is not observed). In particular, the coverage probability for the Weibull shape parameter (with true valueγ=1) is near‐nominal for all study designs. Because the exponential distribution is not a special case of the log‐logistic distribution, the log‐logistic model is misspecified. There is no true value for the log‐logistic log shapelnγ, and$\ln\lambda_{0}$ has a different interpretation in the two models. Nonetheless, the coverage probabilities for$\beta_{\text{sus}}$ are near‐nominal for all study designs, and the coverage probabilities for$\beta_{\inf}$ are near‐nominal for all study designs when who‐infected‐whom is observed and for the valid study designs when who‐infected‐whom is not observed. The log‐logistic model, with both rate and shape parameters, has more flexibility to mimic the exponential distribution than vice versa.

**TABLE 2:** 95% confidence interval coverage probabilities for pairwise AFT models fit to exponential contact intervals.

Model	Study design	$\beta_{\text{sus}}$	$\beta_{\inf}$	$\ln\lambda_{0}$	$\ln\gamma_{\text{int}}$	$\ln\mu_{0}$
**Who‐infected‐whom observed**						
Log‐logistic	Complete cohort	0.934	0.948	0.748	—	0.956
	CT with delayed entry	0.954	0.947	0.749	—	0.959
	*CT without delayed entry*	0.957	0.947	0.745	—	0.000
	*Ignoring external infection*	0.952	0.947	0.763	—	—
Weibull	Complete cohort	0.933	0.945	0.942	0.946	0.954
	CT with delayed entry	0.951	0.944	0.939	0.950	0.958
	*CT without delayed entry*	0.954	0.946	0.940	0.947	0.000
	*Ignoring external infection*	0.951	0.946	0.933	0.948	—
**Who‐infected‐whom not observed**						
Log‐logistic	Complete cohort	0.933	0.950	0.801	—	0.837
	CT with delayed entry	0.954	0.963	0.817	—	0.929
	*CT without delayed entry*	0.948	0.830	0.128	—	0.195
	*Ignoring external infection*	0.945	0.782	0.073	—	—
Weibull	Complete cohort	0.930	0.956	0.925	0.940	0.785
	CT with delayed entry	0.949	0.959	0.946	0.952	0.930
	*CT without delayed entry*	0.950	0.793	0.583	0.949	0.196
	*Ignoring external infection*	0.953	0.749	0.470	0.951	—

*Note*: The flawed study designs are in italics.

Table 3 shows coverage probabilities from pairwise AFT models with exponential and Weibull internal contact intervals analyzing simulated data generated with log‐logistic internal contact intervals. Because the log‐logistic distribution is not a special case of the Weibull distribution, both of these models are misspecified. The exponential model produced low coverage probabilities when who‐infected‐whom was observed and a mixture of low and abnormally high coverage probabilities (suggesting high variance) when who‐infected‐whom was not observed. The Weibull model produced near‐nominal coverage probabilities for$\beta_{\text{sus}}$ and$\beta_{\inf}$ under the valid study designs whether or not who‐infected‐whom was observed. Under the flawed study designs, it produced near‐nominal coverage probabilities for$\beta_{\text{sus}}$ but substantially lower coverage probabilities for$\beta_{\inf}$. The Weibull model produced consistently low coverage probabilities for$\ln\lambda_{0}$ and$\ln\gamma_{\text{int}}$, which have different interpretations in the log‐logistic and Weibull distributions.

**TABLE 3:** 95% confidence interval coverage probabilities for pairwise AFT models fit to log‐logistic contact intervals.

Model	Study design	$\beta_{\text{sus}}$	$\beta_{\inf}$	$\ln\lambda_{0}$	$\ln\gamma_{\text{int}}$	$\ln\mu_{0}$
**Who‐infected‐whom observed**						
Exponential	Complete cohort	0.693	0.645	0.000	—	0.854
	CT with delayed entry	0.637	0.646	0.000	—	0.928
	*CT without delayed entry*	0.664	0.645	0.000	—	0.000
	*Ignoring external infection*	0.624	0.645	0.000	—	—
Weibull	Complete cohort	0.916	0.922	0.034	0.013	0.911
	CT with delayed entry	0.912	0.924	0.043	0.015	0.959
	*CT without delayed entry*	0.906	0.923	0.045	0.015	0.000
	*Ignoring external infection*	0.913	0.923	0.044	0.016	—
**Who‐infected‐whom not observed**						
Exponential	Complete cohort	0.687	0.894	0.000	—	0.860
	CT with delayed entry	0.621	0.972	0.000	—	1.000
	*CT without delayed entry*	0.620	0.972	0.000	—	0.898
	*Ignoring external infection*	0.620	0.972	0.000	—	—
Weibull	Complete cohort	0.935	0.936	0.077	0.072	0.918
	CT with delayed entry	0.936	0.919	0.119	0.105	0.991
	*CT without delayed entry*	0.935	0.894	0.118	0.004	0.897
	*Ignoring external infection*	0.936	0.894	0.118	0.004	—

*Note*: The flawed study designs are in italics.

Because so little is known about infectiousness profiles of communicable diseases, a parametric family for the contact interval distribution will usually need be chosen empirically. Table 4 shows that the correctly‐specified model usually had the lowest Akaike information criterion (AIC) [49], suggesting that the choice of a parametric model can be guided by the AIC or other measures of model fit.

**TABLE 4:** Proportion of fitted models with the lowest Akaike information criterion (AIC) under valid epidemiologic study designs.

Contact intervals	Study design	Exponential	Weibull	Log‐logistic
**Who‐infected‐whom observed**				
Exponential	Complete cohort	0.727	0.105	0.168
	CT with delayed entry	0.734	0.104	0.162
Log‐logistic	Complete cohort	0.000	0.058	0.942
	CT with delayed entry	0.000	0.059	0.942
**Who‐infected‐whom not observed**				
Exponential	Complete cohort	0.728	0.112	0.161
	CT with delayed entry	0.734	0.110	0.157
Log‐logistic	Complete cohort	0.000	0.059	0.942
	CT with delayed entry	0.000	0.059	0.941

## 4 Los Angeles County Influenza a (H1N1) Data

To give an example of pairwise AFT modeling of infectious disease transmission data, we analyze influenza A (H1N1) household surveillance data collected by the Los Angeles County Department of Public Health (LACDPH) in April and May, 2009. The data was collected using the following protocol [50]:

1. Between April 14 and May 18, nasopharyngeal swabs and aspirates were taken from individuals who reported to the LACDPH or other local health care providers with acute febrile respiratory illness (AFRI), defined as a fever$\geq 37.8^{\circ }C$ plus at least one of cough, sore throat, or rhinorrhea (runny nose). These specimens were tested for influenza using reverse transcriptase polymerase chain reaction (RT‐PCR).
2. Patients whose specimens tested positive for pandemic influenza A (H1N1) or for influenza A of undetermined subtype were invited to participate in a phone interview. These interviews used a standard questionnaire developed by the LACDPH to collect information about his or her household contacts, including sex, age, and antiviral prophylaxis use. For index cases under 18 years of age, an adult proxy was interviewed.
3. The initial interview and, when necessary, a follow‐up interview were used to obtain the symptom onset dates of AFRI episodes in the household up to 14 days after the symptom onset date of the index case. All interviews were completed between April 30 and June 1.

For simplicity, we assume all AFRI episodes among household members were caused by influenza A (H1N1). All index cases are assumed to be external infections, and all other household members are assumed to be at risk of infection from both household members and external sources. This study design corresponds to contact tracing with delayed entry in the simulation study above.

The primary analysis assumed an incubation period of 2 days, a latent period of 0 days, and an infectious period of 6 days. These natural history assumptions are adapted from Yang et al. [51]. Households were identified upon clinical presentation of an index case, so household members were considered to be at risk of infection from the infection time of the index case (which depends on the assumed incubation period) until 14 days after the infection time of the index case. In a sensitivity analysis, we varied the assumed latent and infectious periods.

The covariates used in our analysis were sex (male=1 for males andmale=0 for females), age category (adult=1 for ages≥18 years andadult=0 otherwise), and antiviral prophylaxis. Antiviral prophylaxis was assumed to be initiated on the day following the symptom onset of the index case in each household, so it was handled as a time‐dependent covariate. Each pair had covariate values for the infectious individual (male_inf,adult_inf, andproph_inf) and for the susceptible individual (male_sus,adult_sus, andproph_sus). In external pairs, all infectiousness covariates were set to zero. We considered exponential, Weibull, and log‐logistic distributions for the internal and external contact interval distributions. All models were fit using the Broyden, Fletch, Goldfarb, and Shanno algorithm (BFGS in theR function optim) with starting parameter values taken from an initial fit using exponential internal and external contact intervals.

Statistical analysis was conducted inR version 4.4.1 (https://www.r‐project.org) using TranStat version 0.3.7 (https://www.gihub.com/ekenah/TranStat). The data set and analysis code (with instructions on use) are available in the Supporting Information.

### 4.1 Data Analysis Results

The household data collected by the Los Angeles County Department of Public Health included 299 individuals in 58 households. There were 99 probable influenza infections, of which 62 were index cases—four households had co‐primary cases with symptom onsets on the same day. There were three people missing data on sex, four people missing data on age, and 56 people missing data on antiviral prophylaxis. The 62 individuals with missing data came from 17 households with 36 infections, of which 19 were index cases. Because we assume all household members can infect or be infected by other household members, we excluded the entire household if any of its members was missing data. In the complete‐cases data set, we have 41 households with 63 infections, of which 43 were index cases.

Using the complete‐cases data set, we fit a model with main effects for all six covariates using all nine possible combinations of internal and external contact interval distributions. All models were fit using the BFGS algorithm for optimization as in the simulations, which is the default in TranStat. Table 5 shows the resulting AIC values. The three minimum AIC values occur for exponential internal contact intervals. Among these three, the lowest AIC occurs for log‐logistic external contact intervals. Using exponential internal contact intervals and log‐logistic external contact intervals, we built a model using backwards selection to achieve the minimum AIC. This removed all covariates except for three: age category for infectiousness (adult_inf), age category for susceptibility (adult_sus), and prophylaxis by susceptibles (proph_sus). The AIC of this model was 203.59.

**TABLE 5:** AIC values for regression models including all available covariates.

	External contact intervals		
Internal contact intervals	Exponential	Weibull	Log‐logistic
Exponential	207.74	208.01	207.66
Weibull	209.25	209.87	209.58
Log‐logistic	209.22	209.82	209.52

We then checked for external interaction terms, which allow a covariate to have different coefficients in the internal and external transmission models. An external interaction term withadult_sus had a *p*‐value of 0.89 and increased the AIC to 205.57. An external interaction term withproph_sus had a *p*‐value of 0.87 but reduced the AIC to 202.37. A joint likelihood ratio test for the main effect and external interaction term forproph_sus in this model yielded a *p*‐value of 0.008, which is consistent with the *p*‐value of 0.012 forproph_sus in the model with no interaction term. For simplicity, we did not keep this interaction term in the model.

Our final model is summarized at the top of Table 6. The coefficients for covariates are log rate ratios,$\text{intercept}=\ln\lambda_{0}$ (the log baseline internal rate parameter),$\text{xintercept}=\ln\mu_{0}$ (the log baseline external rate parameter), and$\text{xlogshape}=\ln\gamma_{\text{ext}}$ (the external log‐logistic shape parameter). The BFGS algorithm did not find an upper 95% likelihood ratio confidence limit foradult_inf, so we calculated this upper limit by refitting the model using the Nelder‐Mead algorithm. Both algorithms produced nearly identical point and interval estimates for all other parameters in the model.

**TABLE 6:** Coefficient estimates from final model with likelihood ratio confidence limits and *p*‐values.

Coefficient	Estimate	95% confidence interval	*p*‐value
**Accounting for external infection**			
Intercept	−4.90	(−22.92, −3.38)	<0.001
Adult (infectiousness)	1.36	(−0.36, 6.47)	0.131
Adult (susceptibility)	−0.48	(−1.46, 0.18)	0.138
Prophylaxis (susceptibility)	−1.06	(−2.26, −0.28)	0.012
External intercept	−4.10	(−6.04, −3.59)	0.021
External log shape	0.80	(−0.76, 1.50)	0.191
**Ignoring external infection**			
Intercept	−4.10	(−5.46, −3.04)	<0.001
Adult (infectiousness)	0.76	(−0.45, 2.10)	0.218
Adult (susceptibility)	−0.77	(−1.87, 0.37)	0.178
Prophylaxis (susceptibility)	−0.83	(−2.14, 0.30)	0.155

The model suggests that adults were more infectious and less susceptible than children, but the small number of transmission events observed makes these results inconclusive. The predicted rate ratio for susceptibility associated with antiviral prophylaxis is 0.34(0.10,0.76), so the model strongly suggests that antiviral prophylaxis in susceptibles reduced their risk of infection. We found no clear evidence of differences in infectiousness or susceptibility by sex, and we found no clear evidence of an effect of antiviral prophylaxis on infectiousness.

Table 7 shows the predicted household SAR by the age of the infectious individual, the age of the susceptible individual, and antiviral prophylaxis in the susceptible individual. The higher infectiousness and lower susceptibility of adults is apparent, as is the protective effect of antiviral prophylaxis. Because the predicted household SAR depends on multiple parameters in the regression model, we used Wald confidence intervals.

**TABLE 7:** Predicted household secondary attack risks with Wald confidence intervals.

Transmission			
From	To	Estimate	95% confidence interval
Child	Child untreated	4.4%	(0.5%, 34.6%)
	Child on prophylaxis	1.5%	(0.2%, 13.7%)
Child	Adult untreated	2.7%	(0.3%, 20.1%)
	Adult on prophylaxis	0.9%	(0.1%, 7.9%)
Adult	Child untreated	15.9%	(6.4%, 36.5%)
	Child on prophylaxis	5.8%	(2.0%, 15.9%)
Adult	Adult untreated	10.2%	(4.3%, 22.8%)
	Adult on prophylaxis	3.6%	(1.2%, 10.5%)

To see how accounting for external sources of infection affected our analysis, we re‐fit our final model using only data on infectious‐susceptible pairs within households. This model is summarized at the bottom of Table 6. The two models give similar results, but accounting for external infection gave us greater statistical power to estimate the effects of age and antiviral prophylaxis. Using a two‐parameter contact interval distribution did not restore the statistical power lost by ignoring external sources of infection. Refitting the final model without external rows using Weibull or log‐logistic contact intervals instead of exponential contact intervals yielded a *p*‐value of 0.160 for the coefficient on antiviral prophylaxis in susceptible individuals.

Table 8 shows the results of a sensitivity analysis where we varied assumptions about the latent and infectious periods. The infectiousness rate ratio for age category and its *p*‐value are highly sensitive to the assumed latent and infectious periods. The susceptibility rate ratio for age category and its *p*‐value are somewhat more stable. The susceptibility rate ratio for antiviral prophylaxis and its *p*‐value are remarkably stable. The rate ratio varies from 0.35 to 0.41, and its *p*‐value varies from 0.011 to 0.026. The loss of statistical power when we fail to account for external sources of infection is consistent throughout the sensitivity analysis.

**TABLE 8:** Log rate ratios with likelihood ratio confidence intervals and *p*‐values from sensitivity analysis.

	Accounting for external infection			Ignoring external infection		
Coefficient	Estimate	95% CI	*p*‐value	Estimate	95% CI	*p*‐value
**Latent period = 1 day**						
Adult (infectiousness)	0.08	(−1.24, 1.37)	0.894	0.06	(−0.99, 1.09)	0.909
Adult (susceptibility)	−0.63	(−1.52, 0.07)	0.074	−0.58	(−1.61, 0.52)	0.286
Prophylaxis (susceptibility)	−1.05	(−2.26, −0.23)	0.013	−0.95	(−2.24, 0.13)	0.087
**Infectious period = 5 days**						
Adult (infectiousness)	1.73	(−0.43, 4.96)	0.140	0.58	(−0.68, 1.95)	0.366
Adult (susceptibility)	−0.41	(−1.40, 0.20)	0.167	−0.91	(−2.08, 0.25)	0.121
Prophylaxis (susceptibility)	−1.02	(−2.18, −0.29)	0.011	−0.69	(−2.02, 0.47)	0.246
**Infectious period = 7 days**						
Adult (infectiousness)	0.29	(−0.99, 1.73)	0.649	0.20	(−0.88, 1.28)	0.707
Adult (susceptibility)	−0.57	(−1.38, 0.15)	0.103	−0.44	(−1.44, 0.64)	0.406
Prophylaxis (susceptibility)	−0.90	(−1.96, −0.12)	0.026	−0.73	(−1.89, 0.30)	0.167

## 5 Discussion

Our simulation results showed that the pairwise AFT model produces reliable point and interval estimates of parameters for the internal and external contact interval distributions when correctly specified. In particular, it produced reliable estimates of rate ratios for infectiousness and susceptibility under valid epidemiologic study designs whether or not who‐infected‐whom was observed. When who‐infected‐whom was observed, these rate ratio estimates were surprisingly robust to flawed epidemiologic study design. Estimates of rate ratios for infectiousness were more sensitive to both observation of who‐infected‐whom and epidemiologic study design than estimates of rate ratios for susceptibility. A sufficiently flexible pairwise AFT model can accurately estimate rate ratios even when it is slightly misspecified (e.g., a Weibull model used for data generated with log‐logistic contact intervals). It is likely that the simulation results would have been even better if we had used likelihood ratio confidence intervals or taken steps to identify a good starting point for maximization of the likelihood.

There are several limitations of the LACDPH household data analysis that point toward future research topics. In the interest of simplicity, the handling of missing data was crude. We removed entire households when any member was missing a covariate, and we assumed fixed incubation, latent, and infectious periods to avoid treating these times as missing. Multiple imputation or data‐augmented Bayesian methods [52] would be a more principled way to handle missing data, but their implementation needs to account for the dependencies induced by disease transmission. With no clear scientific basis for choosing parametric families for the internal and external contact interval distributions, we compared models using the AIC. Although our simulations suggest this can be a reliable approach when the available parametric families include the true distributions, an extension of the semiparametric model of Kenah [29] that could handle external sources of infection would not require a choice of parametric families. Finally, the pairwise AFT model showed occasional numerical instability. Although we dealt successfully with this problem ad hoc, it deserves more systematic investigation.

The pairwise AFT model can be viewed as an extension of the longitudinal chain‐binomial model [53, 54] to continuous time. Like these models, it accounts for dependent events and for infection from external sources even when who‐infected‐whom is not observed. Unlike these models, it allows flexibility in the infectiousness profile without a large number of nuisance parameters, and it can be specified, fit, and interpreted in a manner similar to standard regression models. The simulation study showed that it produces accurate point and interval estimates when the epidemiologic study design is valid and the chosen parametric models have the flexibility to mimic the true internal and external contact interval distributions. The analysis of the LACDPH influenza A (H1N1) household data showed that the model can produce insights relevant to public health and that accounting for external sources of infection is important. When who‐infected‐whom is observed (which occurs rarely in practice), estimation of rate ratios for susceptibility and infectiousness is remarkably robust to flawed epidemiologic study design. This suggests that pathogen phylogenies, which provide partial information on who‐infected‐whom, could improve precision and reduce bias [55]. With or without pathogen genome sequences, pairwise AFT regression models can generate detailed insights about the transmission of infectious diseases from longitudinal studies of close contact groups or from contact tracing data. These insights can help us design efficient and effective public health interventions to control future epidemics.

## Conflicts of Interest

The authors declare no conflicts of interest.

## Supporting information




**Data S1: pAFTsims.R**: R script to generate epidemic simulations and fit pairwise AFT models.
**pAFTsimulation.py**: Python script for epidemic simulation called by pAFTsims.R.
**results.csv**: Comma‐separated values (CSV) file with simulation results.
**pAFTanalysis.R**: R script to analyze results.csv, which generates Tables 1–4, Figures 2–5, and S1–S4.
**LAdata_2023‐08.csv**: Los Angeles County Department of Public Health household influenza transmission data.
**AFT_LAanalysis.R**: R script for LACDPH household data analysis, which generates Tables 5–8.

**Appendix S2:** Supporting Information.

## Data Availability

The data that supports the findings of this study are available in the supporting information of this article.
