# Supplementary material for: Pairwise Accelerated Failure Time Regression Models for Infectious Disease Transmission in Close‐Contact Groups With External Sources of Infection
Source: Stat Med. 2024 Oct 3;43(27):5138–54. doi: 10.1002/sim.10226 (PMC11583957; doi:10.1002/sim.10226)
Supplement: Supplementary file 2 — Appendix S2: Supporting Information. [file SIM-43-5138-s001.pdf]

Supplementary Figures:
Pairwise accelerated failure time regression models for infectious disease transmission in close-contact groups with external sources of infection

Yushuf Sharker, Zaynab Diallo, Wasiur R. KhudaBukhsh,
and Eben Kenah

May 24, 2024

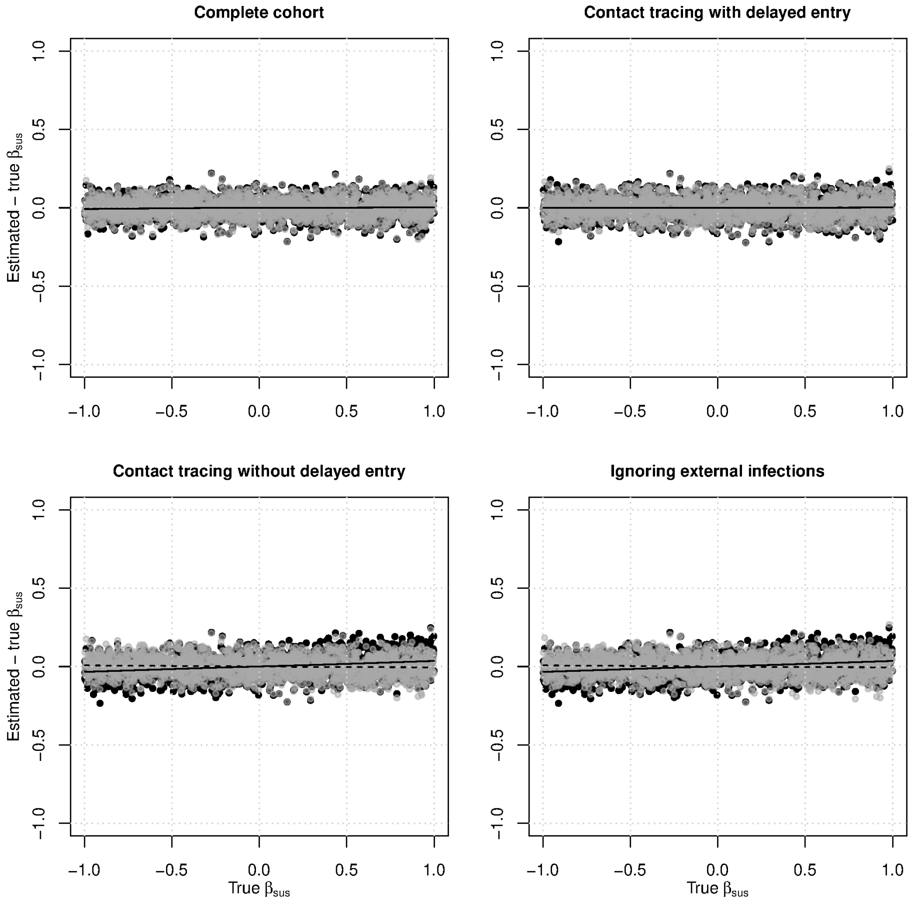

Figure 1: The bias $\hat{\beta}_{\text{sus}} - \beta_{\text{sus}}$ versus the true β_{sus} for correctly-specified log-logistic pairwise AFT models fit to simulated data under all four study designs. Gray dots represent analyses where who-infected whom was observed, and black dots represent analyses where who-infected-whom was not observed. In each plot, the dashed locally-weighted polynomial regression (LOWESS) line represents the smoothed mean of the gray dots, and the solid LOWESS line represents the smoothed mean of the black dots. The dashed lines are sometimes obscured by the solid lines.

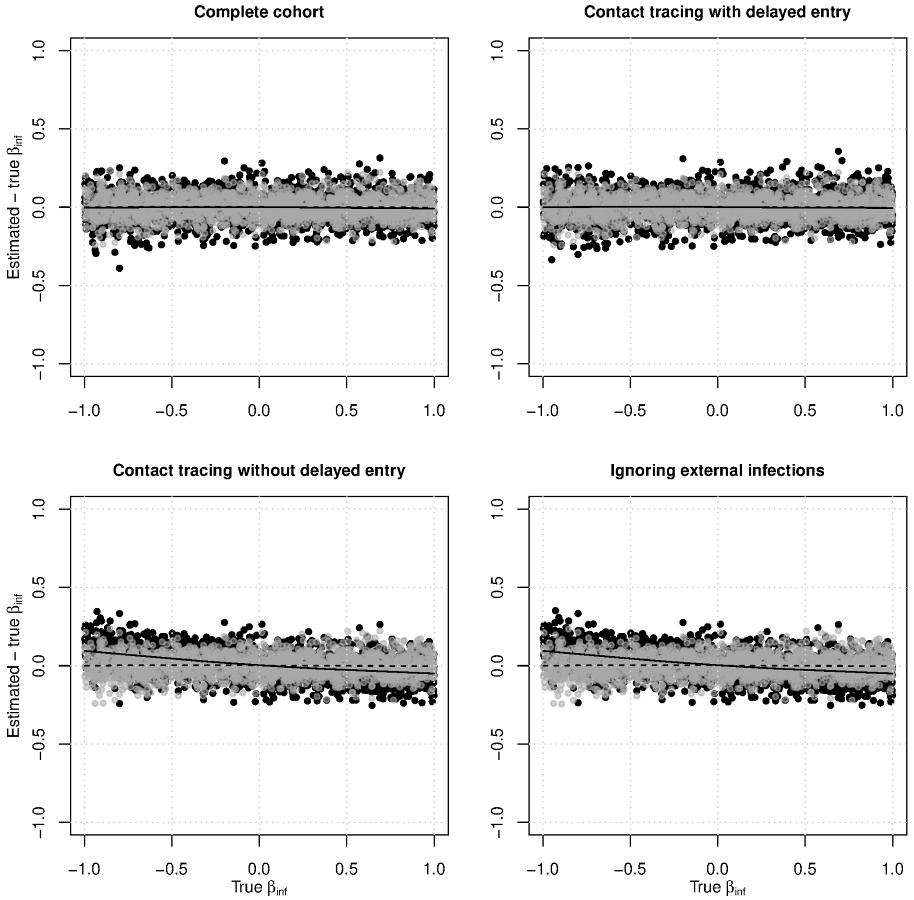

Figure 2: The bias $\hat{\beta}_{\text{inf}} - \beta_{\text{inf}}$ versus the true β_{inf} for correctly-specified log-logistic pairwise AFT models fit to simulated data under all four study designs. Gray dots represent analyses where who-infected whom was observed, and black dots represent analyses where who-infected-whom was not observed. In each plot, the dashed LOWESS line represents the smoothed mean of the gray dots and the solid LOWESS line represents the smoothed mean of the black dots. The dashed lines are sometimes obscured by the solid lines.

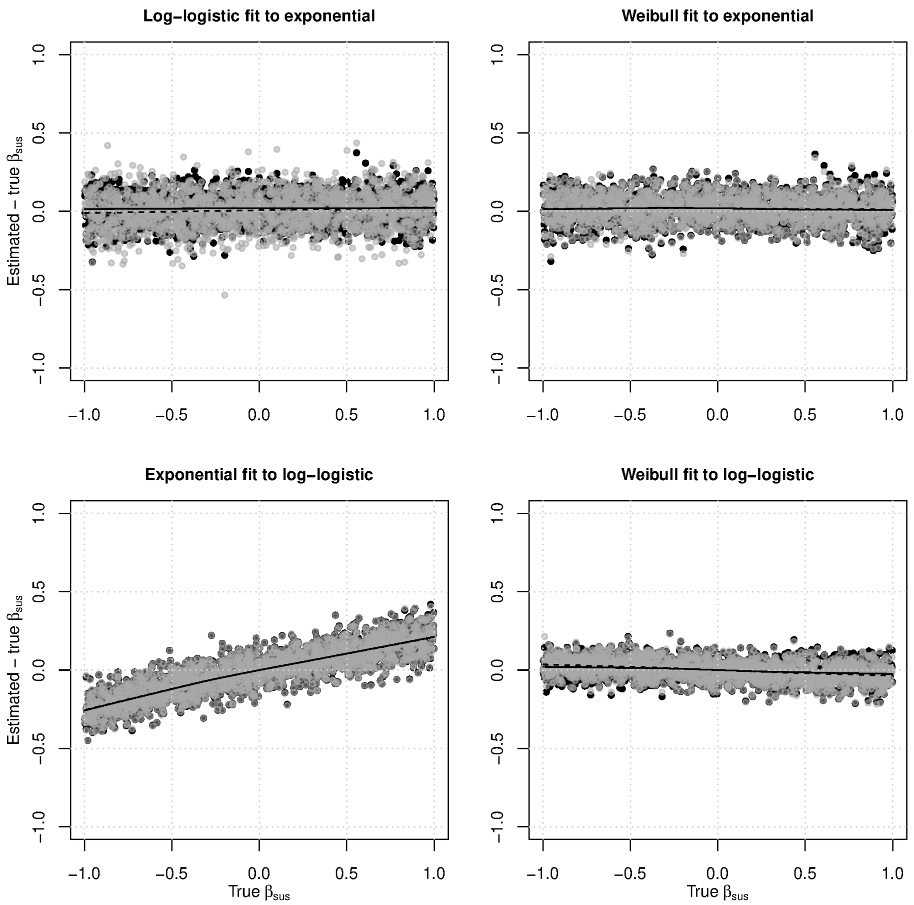

Figure 3: The bias $\hat{\beta}_{\text{sus}} - \beta_{\text{sus}}$ versus the true β_{sus} for pairwise AFT models under the complete cohort study design. In the top two panels, the simulated data was generated using exponential internal contact intervals, so the log-logistic model is misspecified but the Weibull model is correctly specified. In the bottom two panels, the simulated data was generated using log-logistic internal contact intervals, so the exponential and Weibull models are both misspecified. Gray dots represent analyses where who-infected whom was observed, and black dots represent analyses where who-infected-whom was not observed. In each plot, the dashed LOWESS line represents the smoothed mean of the gray dots and the solid LOWESS line represents the smoothed mean of the black dots. The dashed lines are sometimes obscured by the solid lines.

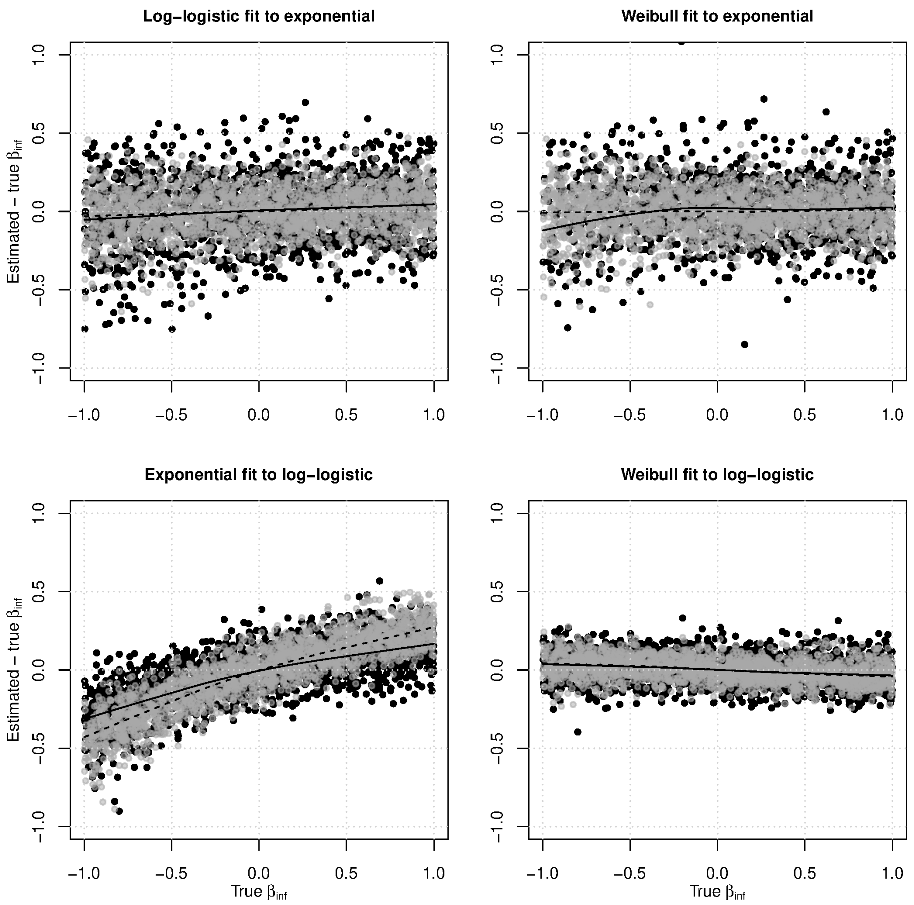

Figure 4: The bias $\hat{\beta}_{\text{inf}} - \beta_{\text{inf}}$ versus the true β_{inf} for pairwise AFT models under the complete cohort study design. In the top two panels, the simulated data was generated using exponential internal contact intervals, so the log-logistic model is misspecified but the Weibull model is correctly specified. In the bottom two panels, the simulated data was generated using log-logistic internal contact intervals, so the exponential and Weibull models are both misspecified. Gray dots represent analyses where who-infected whom was observed, and black dots represent analyses where who-infected-whom was not observed. In each plot, the dashed LOWESS line represents the smoothed mean of the gray dots and the solid LOWESS line represents the smoothed mean of the black dots. The dashed lines are sometimes obscured by the solid lines.
